# Risk factors for steroid-refractory in immune checkpoint inhibitor-induced colitis: a retrospective cohort study

**DOI:** 10.3389/fimmu.2025.1623150

**Published:** 2025-10-07

**Authors:** Ke Meng, Jing Chen, Junzhe Chen, Shengjie Sun, Hui Li, Guanzhou Zhou, Fei Pan

**Affiliations:** ^1^ Department of Gastroenterology and Hepatology, The First Medical Center of Chinese People's Liberation Army (PLA) General Hospital, Beijing, China; ^2^ School of Medicine, Nankai University, Tianjin, China; ^3^ Department of Oncology, The First Medical Center of Chinese People's Liberation Army (PLA) General Hospital, Beijing, China

**Keywords:** immune checkpoint inhibitors, steroid refractoriness, immune checkpoint inhibitor-induced colitis, risk factors, interleukin-6

## Abstract

**Background:**

Immune checkpoint inhibitors (ICIs) represent an effective treatment for various malignant tumors. However, the utilization of ICIs is frequently accompanied by immune-related adverse events (irAEs), among which immune checkpoint inhibitor (ICI)-induced colitis is a notable complication. Current clinical guidelines recommend corticosteroids as the first-line therapy for ICI-induced colitis. Nevertheless, subset of patients fails to respond adequately to corticosteroid therapy, resulting in steroid refractoriness. At present, studies investigating the risk factors for steroid-refractory remain limited.

**Patients and methods:**

A retrospective analysis was conducted on patients diagnosed with ICI-induced colitis after malignant tumor treatment with ICIs. Data collected included demographics, tumor and ICIs types, time to colitis onset, number of ICIs treatments, clinical manifestations (diarrhea, abdominal pain, bloody stool, fever), endoscopic findings (ulcerative lesions, extent of lesion distribution), laboratory results, grades of diarrhea and colitis, and corticosteroid treatment response. Patients were stratified into steroid-responsive and steroid-refractory groups. Multivariate logistic regression analysis was employed to identify risk factors related to steroid-refractory. Kaplan-Meier survival analysis and log-rank tests were conducted to compare survival time differences between the two groups.

**Results:**

A total of 57 patients were included, with 45 patients in the steroid-responsive group and 12 patients in the steroid-refractory group. Univariate analysis revealed differences between the two groups in the time to colitis onset (median days: 97 vs. 141, *P* = 0.037), presence of fever (4.4% vs. 25.0%, *P* = 0.045), presence of ulcerative lesions (26.9% vs. 34.6%, *P* = 0.036), grades of colitis (*P* = 0.011), and serum interleukin-6 (IL-6) level (24.1 ± 20.5 pg/mL vs. 81.7 ± 38.7 pg/mL, *P* < 0.001). Multivariate regression analysis indicated that serum IL-6 level was an independent risk factor for steroid-refractory. Kaplan-Meier survival analysis showed no significant difference in survival time between the two groups.

**Conclusions:**

For patients with ICI-induced colitis, serum IL-6 level at colitis onset could serve as an independent risk indicator for predicting the efficacy of corticosteroid therapy. Early consideration of selective immunosuppressive therapy (SIT) may be warranted with caution for patients with high serum IL-6 level.

## Introduction

Immune checkpoint inhibitors (ICIs) have emerged as a significant breakthrough in the field of oncology in recent years and are now widely applied in the treatment of various malignant tumors, including melanoma, non-small cell lung cancer, renal cell carcinoma, gastric cancer, and hepatocellular carcinoma. These agents enhance T-cell activity by blocking immune checkpoint proteins such as programmed death ligand 1 (PD-L1), programmed cell death protein 1 (PD-1), and cytotoxic T-lymphocyte-associated antigen 4 (CTLA-4), thereby exerting anti-tumor effects (1, 2). However, the expanding use of ICIs has been accompanied by an increasing incidence of immune-related adverse events (irAEs). Among these, immune checkpoint inhibitor (ICI)-induced colitis is one of the most common gastrointestinal toxicities, with clinical manifestations ranging from mild diarrhea to life-threatening colitis with severe complications, such as perforation. The reported incidence of ICI-induced colitis ranges from 1.3% to 13.6% (3). Notably, patients treated with anti-CTLA-4 therapy experience a higher frequency and severity of gastrointestinal irAEs compared to those treated only with anti-PD-1 or anti-PD-L1 monotherapy. Furthermore, combination therapy with two types of ICIs leads to a higher incidence of colitis (4, 5).

For the treatment of ICI-induced colitis, current guidelines recommend corticosteroids as the first-line therapy (6, 7). Corticosteroids alleviate colitis symptoms by suppressing inflammatory cell infiltration and reducing the release of proinflammatory mediators. However, a subset of patients demonstrates inadequate response to corticosteroid therapy, developing as steroid-refractory colitis. For these patients, selective immunosuppressive therapy (SIT), including agents such as infliximab and vedolizumab, has emerged as a second-line treatment option. A study by Abu-Sbeih et al. (8) revealed that early initiation of SIT in ICI-induced colitis, rather than reserving it for cases of corticosteroid failure, provided greater clinical benefit for patients. Early SIT use improved treatment efficacy, shortened the disease duration, and reduced colitis-related hospitalization time and readmission rates. Consequently, timely identification of patients with ICI-induced colitis who are unlikely to respond to corticosteroids, followed by prompt SIT initiation, is crucial for improving symptom control, minimizing disease burden, and optimizing patient outcomes.

Despite the growing clinical experience with ICIs, research on the risk factors associated with steroid-refractory colitis remains limited. This knowledge gap poses a significant challenge to clinical practice, as the inability to identify high-risk patients early can delay the timely initiation of SIT. Moreover, such delays may prolong patient morbidity, increase hospitalization rates, and contribute to unnecessary healthcare burden. Thus, a clearer understanding of predictive factors for steroid-refractory colitis would enable clinicians to stratify patients more effectively, personalize treatment strategies, and intervene earlier with second-line therapies when indicated. In this context, the present study sought to investigate potential risk factors associated with steroid-refractory colitis in patients receiving ICI therapy.

## Materials and methods

### Study design and population

This was a single-center retrospective study. We investigated adult cancer patients who were treated at the First Medical Center of Chinese PLA General Hospital between January 2018 and October 2024. All enrolled patients had received ICIs therapy (anti-PD-1/PD-L1, anti-CTLA-4, or combination) for malignant tumors and were subsequently developed ICI-induced colitis. Patients with colitis caused by other etiologies, such as inflammatory bowel disease, viral or bacterial infections, or graft-versus-host disease, were excluded. We excluded these patients through comprehensive methods, including medical history review, blood and stool pathogen testing, endoscopic assessment, and histological examination where available. Exclusion of other etiologies for patients who did not undergo colonoscopy and histological evaluation required: (1) Negative blood/stool pathogen testing and toxin testing; (2) No prior inflammatory bowel disease and graft-versus-host disease; (3) Symptom onset post-ICI therapy initiation with corticosteroid/SIT response; (4) Absence of chronic gastrointestinal symptoms prior to ICI treatment.

### Ethics approval

The study was approved by the Medical Ethics Committee of Chinese PLA General Hospital (S2023-237-02) and conducted in accordance with the Declaration of Helsinki.

### Information collection and definition

We retrospectively collected patients’ demographic information, oncological data, information related to ICI-induced colitis and treatment details through the electronic medical record system. Prognostic information was obtained via both telephone follow-up and electronic medical record system. During data collection, diarrhea and colitis were defined and graded according to the Common Terminology Criteria for Adverse Events (CTCAE) version 5.0 (6). ICI-induced colitis was defined as the development of diarrhea or colitis symptoms following the initiation of ICI therapy, which required treatment with corticosteroids or SIT for symptom resolution, and/or confirmed by endoscopic or histopathological evaluation. As current guidelines do not mandate endoscopic evaluation for managing ICI-induced colitis, many patients were diagnosed based on clinical history combined with exclusion of alternative etiologies through blood/stool pathogen testing before initiating corticosteroid therapy. The time to colitis onset was defined as the interval from the first administration of ICIs to the appearance of colitis-related symptoms (e.g., diarrhea, abdominal pain, or bloody stool).

### Patients and clinical characteristics

Patient demographic information (age, gender), tumor types, types of ICIs administered (anti-PD-1/PD-L1, anti-CTLA-4, or combination therapy), and laboratory test results at the time of ICI-induced colitis diagnosed were collected. The laboratory parameters collected included results of complete blood count, biochemical profile, coagulation function, and inflammatory marker- interleukin-6 (IL-6). Serum IL-6 level was quantified using chemiluminescence immunoassay within 48 hours of symptom onset. Samples were collected after a minimum fasting period of 8 hours.

### ICI-induced colitis-related information

The symptoms related to ICI-induced colitis were recorded, including diarrhea, abdominal pain, bloody stool, and fever. The number of ICI treatments administered prior to the onset of colitis and the time to onset of colitis were also documented. The severity of diarrhea and colitis was graded according to the Common Terminology Criteria for Adverse Events (CTCAE) version 5.0 from the National Cancer Institute (6). For patients who underwent colonoscopy, endoscopic features of colitis were recorded, including both the extent of lesion distribution and the degree of mucosal damage. The extent of lesion distribution was classified as either pan-colonic involvement or partial colonic involvement (limited to the left-sided colon or right-sided colon), while the degree of mucosal damage was classified as non-ulcerative inflammation (such as mucosal edema, erythema, loss of vascular pattern, and increased friability) or ulcerative lesions.

### Treatment and outcome assessment

In accordance with guidelines for managing irAEs, patients diagnosed with ICI-induced colitis were initially treated with corticosteroids at a dose of 1–2 mg/kg/day, administered either orally or intravenously, depending on the severity of diarrhea/colitis. ICIs were discontinued in patients with grade ≥ 2 diarrhea. Patients who responded to corticosteroid treatment continued oral corticosteroids with gradual tapering. For steroid-refractory cases, SIT (infliximab) was added. Steroid refractoriness was defined as the lack of clinical response following 3–5 days of adequate corticosteroid therapy. Patient survival time was recorded during follow-up, and defined as the interval from the initiation of ICI treatments to death.

### Statistical analysis

Continuous variables were presented as mean ± standard deviation (SD) or as median with interquartile range (IQR) and compared using either the t-test or the Mann-Whitney U test. Categorical variables were presented as frequencies and percentages, with group comparisons performed using the χ² test or Fisher’s exact test. Multivariate logistic regression analysis was used to identify risk factors associated with steroid-refractory colitis in patients with ICI-induced colitis. Variables with *P* < 0.05 in univariate analysis were included in multivariate logistic regression. Backward stepwise selection (likelihood ratio) was used applied, and collinearity was assessed via variance inflation factor (VIF < 5). Kaplan-Meier curves and the log-rank test were employed to compare survival differences between steroid-responsive and steroid-refractory groups. All statistical tests were two-sided, and a *P* < 0.05 was considered statistically significant. Analyses were performed using SPSS 26.0 (IBM Corp., Armonk, NY, USA). Statistical validity was independently verified by a biomedical statistician.

## Results

### Patient’s characteristics

**Figure 1:**
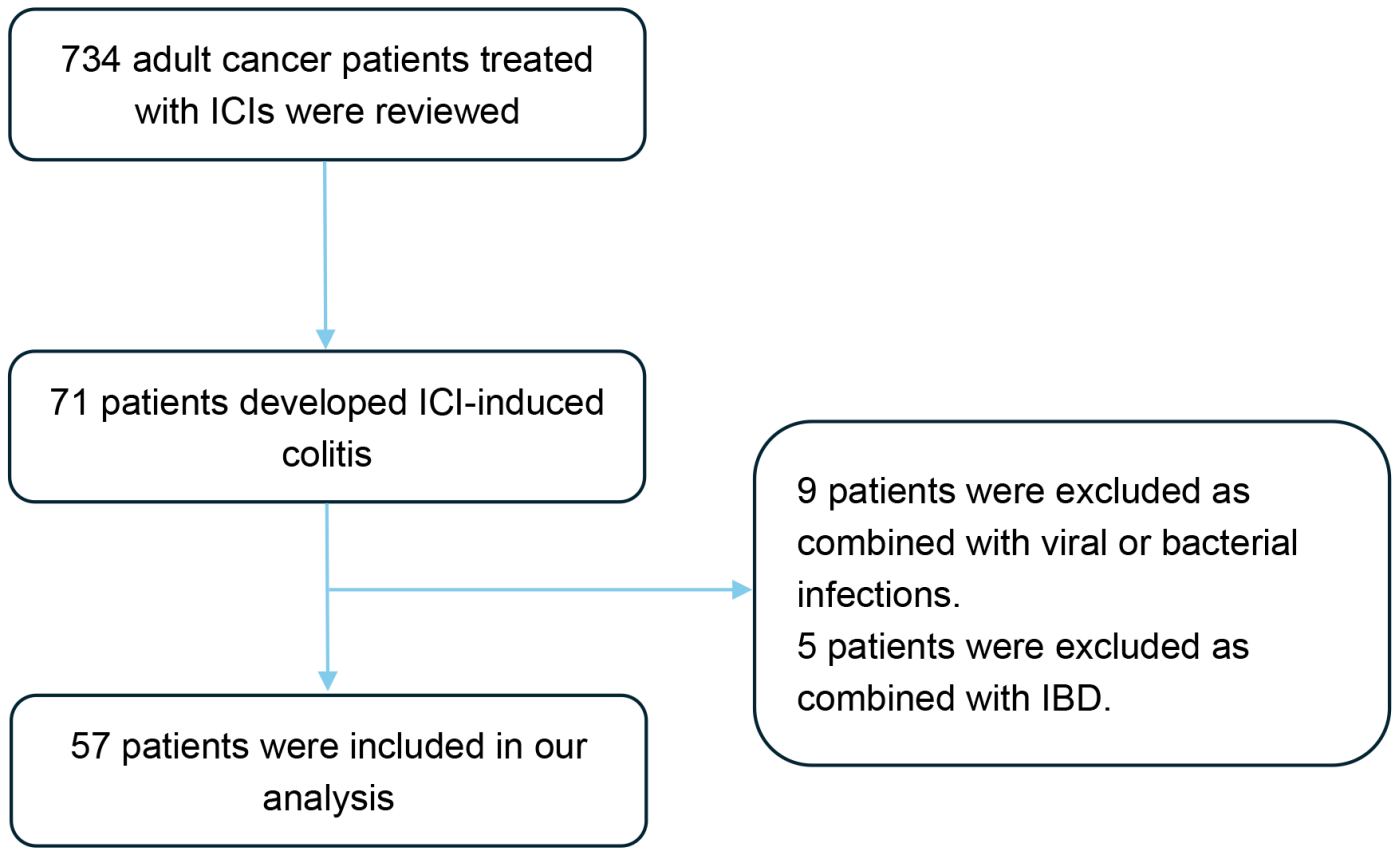
Flow diagram of the study design. A total of 734 patients were screened, of whom 57 patients met the eligibility criteria and were included in the final analysis. ICIs, Immune checkpoint inhibitors; IBD, Inflammatory bowel disease.

A total of 57 patients with ICI-induced colitis were included in this study (**Figure 1**). The patient’s characteristics are presented in **Table 1A**. The cohort comprised 41 males (71.9%) and 16 females (28.1%), with a median age of 60 years (interquartile range [IQR], 54-68). Gastrointestinal cancer represented the most common tumor type (40.4%), followed by lung cancer (26.3%), hepatobiliary cancer (15.8%), and pancreatic cancer (10.5%). Regarding the type of ICI treatment, 41 patients (71.9%) received anti-PD-1 monotherapy, 10 (17.5%) received anti-PD-L1 monotherapy, and 6 (10.5%) received combination therapy with anti-PD-1/PD-L1 and anti-CTLA-4 therapy. Colonoscopy was performed in 26 patients (45.6%). The median follow-up duration was 18 months (range, 2-79).

**Table 1:** Characteristics of patients with ICI-induced colitis.

Characteristics	Values
A. Patient characteristics (n = 57)	
Age, median years (IQR)	60 (54-68)
Gender, n (%)	
Male	41 (71.9)
Female	16 (28.1)
BMI (kg/m^2^, mean ± SD)	22.1 ± 3.1
Tumor types, n (%)	
Gastrointestinal cancer	23 (40.4)
Lung cancer	15 (26.3)
Hepatobiliary cancer	9 (15.8)
Pancreatic cancer	6 (10.5)
Others	4 (7.0)
ICIs types, n (%)	
Anti-PD-1	41 (71.9)
Anti-PD-L1	10 (17.5)
Combination (Anti-PD-1/L1 + Anti-CTLA-4)	6 (10.5)
Months of follow-up, median (IQR)	18 (10-27)
Number of patients underwent colonoscopy (%)	26 (45.6)
B. ICI-induced colitis characteristics (n = 57)	
Time to onset of colitis, median days (IQR)	103 (52-142)
Number of ICI infusions before colitis, median (IQR)	5 (2-8)
Symptoms, n (%)	
Diarrhea	54 (94.7)
Blood in stool	28 (49.1)
Abdominal pain	32 (56.1)
Fever	5 (8.8)
Diarrhea grade, n (%)	
1	7 (12.3)
2	17 (29.8)
3	29 (50.9)
4	4 (7.0)
Colitis grade, n (%)	
1	13 (22.8)
2	39 (68.4)
3	2 (3.5)
4	3 (5.3)
Treatment, n (%)	
Steroid only	45 (78.9)
Steroid + Infliximab	12 (21.1)
C. Endoscopic features, n (%) (n = 26)	
Ulcerative lesions	16 (61.5)
Non-ulcerative inflammation	10 (38.5)
Pan-colonic involvement	16 (61.5)
Partial colonic involvement	10 (38.5)

ICI-induced colitis, Immune checkpoint inhibitor-induced colitis; IQR, interquartile range; SD, Standard deviation; BMI, Body mass index; ICIs, Immune checkpoint inhibitors; PD-1, Programmed cell death protein 1; PD-L1, Programmed death ligand 1; CTLA-4, Cytotoxic T-lymphocyte-associated antigen 4.

### ICI-induced colitis-related information

**Figure 2:**
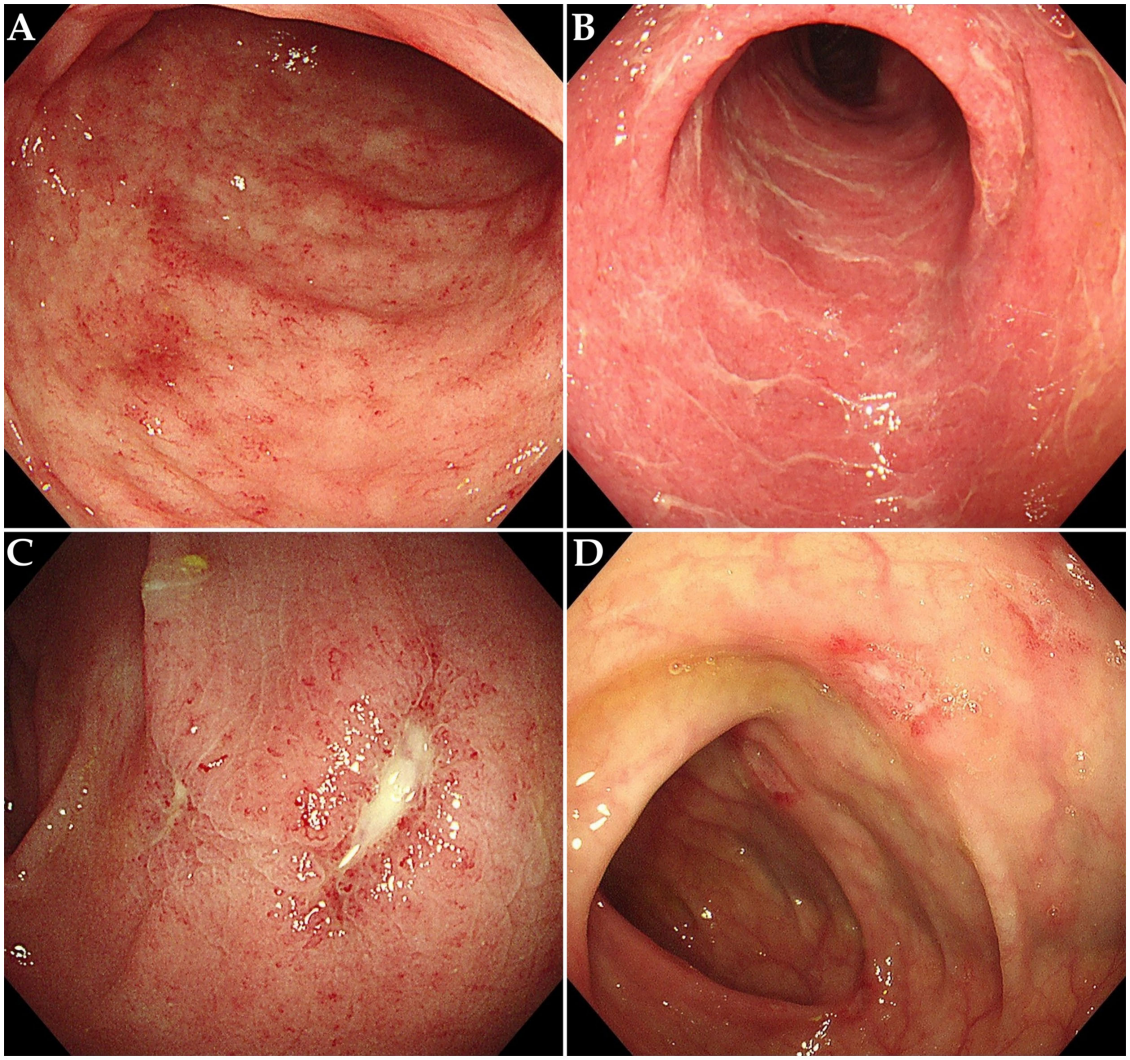
Endoscopic features in patients with ICI-induced colitis. **(A)** Diffuse mucosal erythema with increased friability. **(B)** Mucosal edema, exudate and loss of vascular pattern. **(C, D)** Multiple ulcerative lesions.

The characteristics of ICI-induced colitis are presented in **Tables 1B, C**. The median time to onset of colitis was 103 days (interquartile range [IQR] 52–142 days). The median number of ICI treatments received by patients before the onset of colitis was 5 (IQR 2-8). Diarrhea was the predominant symptom (n = 54, 94.7%), with many patients also experiencing abdominal pain (n = 32, 56.1%) or bloody stool (n = 28, 49.1%). Fever was observed in 5 patients (8.8%) at diagnosis. Among all patients, the number of cases with grade 2, 3, and 4 diarrhea was 17 (29.8%), 29 (50.9%), and 4 (7.0%), respectively. The number of cases with grade 2, 3, and 4 colitis was 39 (68.4%), 2 (3.5%), and 3 (5.3%), respectively. It is important to note that the grades of diarrhea and colitis may not be entirely consistent in many patients with ICI-induced colitis. Among the 26 patients who underwent colonoscopy, 10 patients (38.5%) showed non-ulcerative inflammation (such as mucosal edema, erythema, loss of vascular pattern, or increased friability, **Figures 2A, B**), while 16 patients (61.5%) presented with ulcerative lesions (**Figures 2C, D**). In terms of distribution, pan-colonic involvement was observed in 16 patients (61.5%), whereas partial colonic involvement (confined to either the left or right colon) was observed in 10 patients (38.5%).

### Treatment and outcomes of ICI-induced colitis

In this study, all patients received corticosteroid therapy as initial treatment. Symptomatic remission was achieved in 45 patients (78.9%) (defined as steroid-responsive group), while 12 patients (21.1%) exhibited steroid-refractory colitis (defined as steroid-refractory group), characterized by insufficient response to corticosteroids. These steroid-refractory cases subsequently achieved symptom resolution after receiving adjunctive therapy with infliximab.

### Multivariate analysis and survival analysis

**Figure 3:**
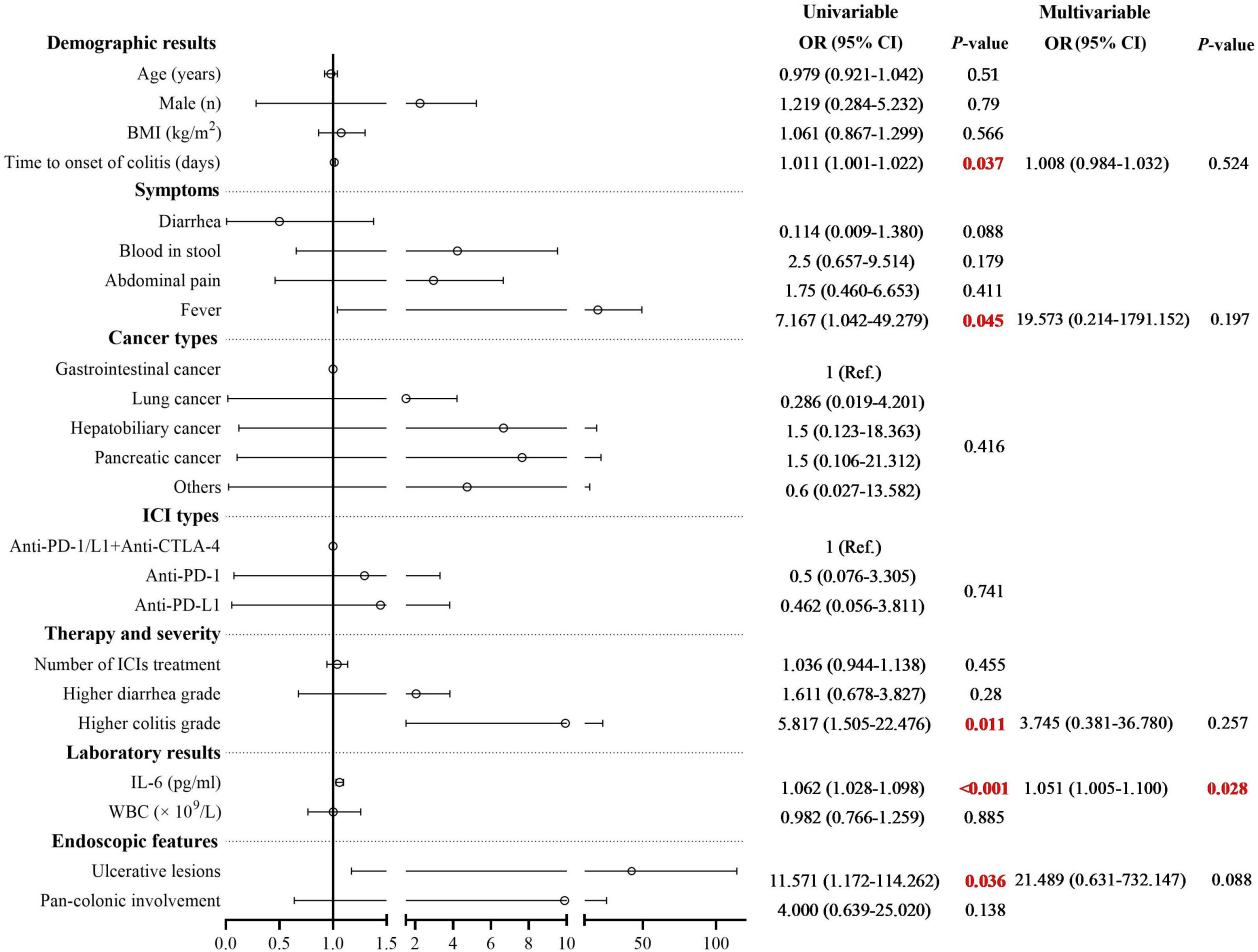
Univariate and multivariate logistic regression analysis of risk factors associated with steroid-refractory colitis. Multivariate analysis revealed that elevated serum IL-6 level was an independent risk factor for poor response to corticosteroid therapy in patients with ICI-induced colitis (OR: 1.051, 95% CI: 1.005-1.100, *P* = 0.028).

**Figure 4:**
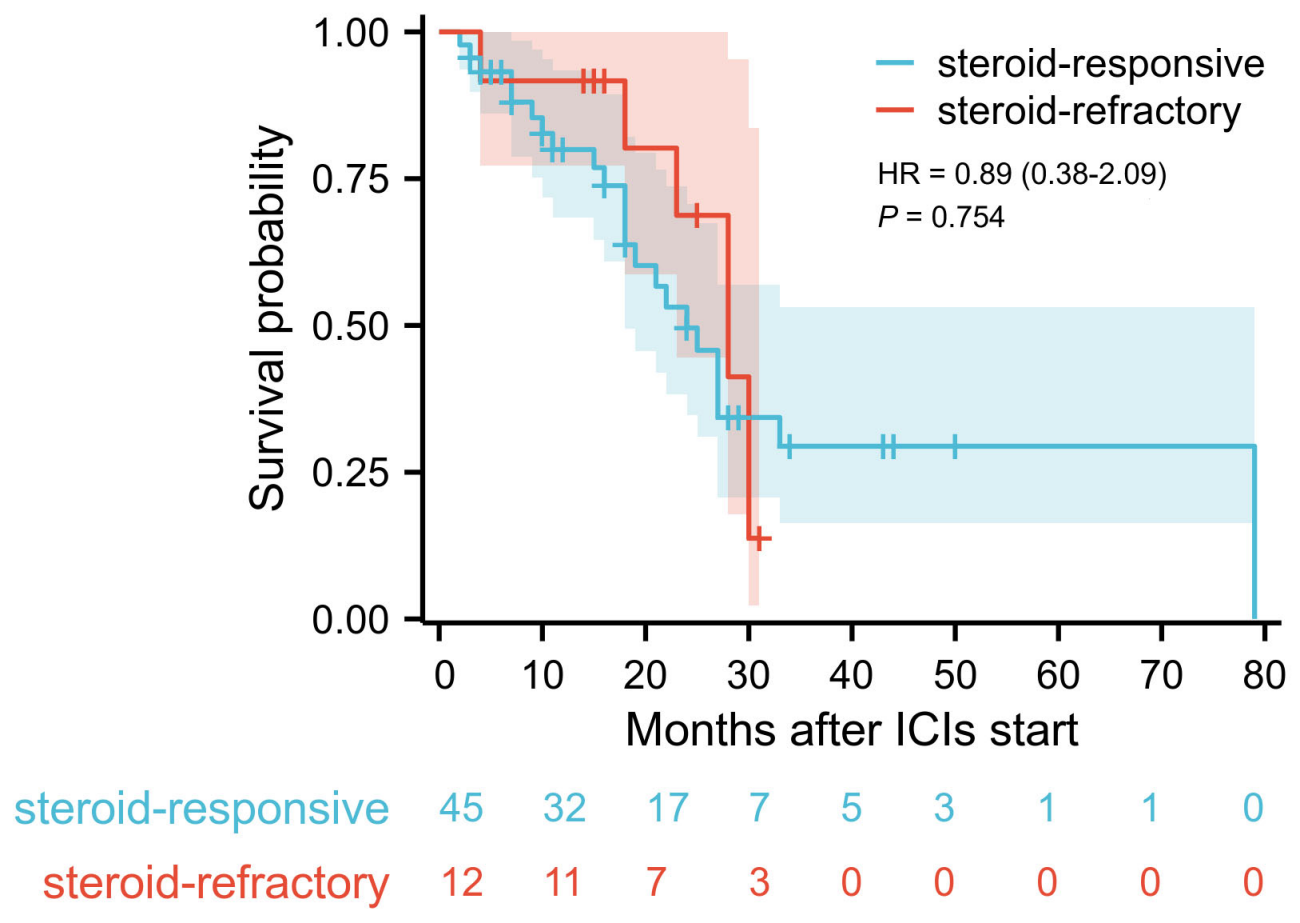
Kaplan-Meier survival curve of overall survival in steroid-responsive group and steroid-refractory group. Blue curve demonstrates the overall survival for steroid-responsive group (n=45), red curve for steroid-refractory group (n=12). Log-rank test revealed no significant difference in survival time (*P* = 0.754).

Multivariate logistic regression analysis was performed to identify risk factors associated with poor response to corticosteroid therapy. Univariate analysis indicated that longer time to onset of colitis (OR: 1.011, 95% CI: 1.001-1.022, *P* = 0.037), presence of fever (OR: 7.167, 95% CI: 1.042-49.279, *P* = 0.045), presence of ulcerative lesions (OR: 11.571, 95% CI: 1.172-114.262, *P* = 0.036), higher colitis grade (OR: 5.817, 95% CI: 1.505-22.476, *P* = 0.011), and elevated serum IL-6 level (OR: 1.062, 95% CI: 1.028-1.098, *P* < 0.001) was associated with poor response to corticosteroid therapy (**Supplementary Table 1**). Multivariate analysis revealed that elevated serum IL-6 level was an independent risk factor for poor response to corticosteroid therapy in patients with ICI-induced colitis (OR: 1.051, 95% CI: 1.005-1.100, *P* = 0.028) (**Figure 3**). During the study period, a total of 30 patients died, corresponding to an overall mortality rate of 52.6%. Among these deaths, 24 patients were attributed to tumor progression, 4 patients were due to cardiovascular disease, and 2 patients were due to pulmonary infection. Kaplan-Meier survival analysis revealed no significant difference in overall survival time between the steroid-responsive group (median 24.0 months, 95% CI: 19.3-28.7) and the steroid-refractory group (median 28.0 months, 95% CI: 21.7-34.3) (*P* = 0.754) (**Figure 4**).

## Discussion

ICIs have achieved remarkable efficacy in the management of various malignant tumors but their use is frequently accompanied by irAEs, among which ICI-induced colitis is one of the relatively common irAEs. Currently, corticosteroids are widely used as the standard first-line therapy for ICI-induced colitis (6). However, approximately 42% of patients exhibit steroid-refractory according to a systematic meta-analysis (9), indicating poor response to corticosteroid therapy in a significant patient population. For these patients, early escalation to SIT, such as infliximab or vedolizumab, can shorten the disease duration and reduce the overall disease burden (8). Therefore, the early identification of risk factors associated with steroid-refractory colitis and timely optimization of therapeutic strategies are critical for optimizing patient outcomes.

This study investigated potential risk factors associated with poor response to corticosteroid therapy in patients with ICI-induced colitis. Univariate analysis revealed that longer time to onset of colitis, presence of fever, ulcerative lesions detected by colonoscopy, higher colitis grade, and elevated serum IL-6 level was associated with poor response to corticosteroid therapy. Multivariate logistic regression analysis demonstrated that elevated serum IL-6 level was independently associated with poor response to corticosteroid therapy in patients with ICI-induced colitis.

IL-6 is a pleiotropic cytokine that plays a crucial role in regulating the activation, proliferation, and differentiation of immune cells. It plays a pivotal role in diverse inflammatory disorders. Elevated serum IL-6 level is associated with a hyper-inflammatory state driven by excessive T-cell activation and cytokine release (10, 11). In ICI-induced colitis, IL-6 promotes the differentiation of Th17 cells and inhibits regulatory T cells (Tregs), thereby exacerbating intestinal inflammatory responses. Zhang et al. (12) reported significantly elevated serum IL-6 concentrations during irAEs, supporting its potential utility as a predictive biomarker for irAEs. Furthermore, Wang et al. (13) revealed that high level of IL-6 is associated with the development of ICI-induced colitis in patients with advanced gastrointestinal cancer receiving ICIs therapy. Therefore, we hypothesize that elevated serum IL-6 level may reflect the severity of intestinal inflammation and magnitude of the systemic immune response, which could further compromise corticosteroid responsiveness.

In our study, univariate analysis also revealed that the presence of ulcerative lesions detected by colonoscopy was associated with poor response to corticosteroid therapy, suggesting that ulcerative lesions may also be a potential predictor of corticosteroid efficacy. This finding is consistent with previous reports demonstrating that the severity of endoscopic mucosal lesions is closely related to both the clinical severity and treatment outcomes in patients with ICI-induced colitis. Specifically, the presence of ulcers and extensive colonic involvement (pancolitis) have been linked to more severe symptoms and reduced responsiveness to corticosteroid therapy (14–17). While endoscopic evaluation of the colonic mucosa inflammation provides a valuable method for assessing ICI-induced colitis severity and predicting therapeutic response, its clinical utility is limited by the invasive nature of the procedure and associated risks. In this study, only 45.6% of the patients underwent colonoscopy. Some patients had severe comorbidities (e.g., coagulopathy, hemodynamic instability) precluding endoscopy. Although the absence of endoscopic data may restrict the generalizability of ulcer-related findings, it is important to note that many patients are unable to undergo colonoscopy in a timely manner. However, clinical management should not be delayed while awaiting endoscopic confirmation. In this regard, serum-based predictive markers provide significant advantages in terms of their accessibility, invasiveness and timeliness.

For patients with steroid-refractory colitis, the use of SIT is currently established as the standard second-line treatment. However, the combined immunosuppressive effects of corticosteroids and SIT must be carefully considered in ICI-induced colitis patients due to potential toxicities, including serious infections such as Pneumocystis jirovecii pneumonia and infliximab-related hypersensitivity reactions (18, 19). Despite these risks, most studies investigating ICIs in advanced malignant tumors demonstrate no detrimental impact of SIT on cancer survival outcomes (20–22). Consistent with these findings, our study also showed no significant difference in survival time between patients treated solely with corticosteroids and those who received infliximab due to poor corticosteroid response during the follow-up period, as indicated by Kaplan-Meier survival analysis. It is important to recognize that survival in patients with malignancies is influenced by multiple factors, including tumor type, disease stage, underlying comorbidities, and responsiveness to anti-cancer therapies. Previous studies have shown that severe irAEs during ICI therapy and poor therapeutic responses to corticosteroids or infliximab may reflect heightened immune activation, potentially correlating with favorable tumor outcomes (23). Collectively, these observations imply that early escalation to SIT with agents like infliximab in steroid-refractory cases may not compromise survival outcomes. Instead, timely SIT implementation could shorten the disease course of ICI-induced colitis, thereby positively influencing quality of life and reducing disease burden.

Our study also has several limitations. First, it was a single-center retrospective study with a relatively small sample size, which may have introduced selection bias. Due to the retrospective nature of the study, some data may be missing or incomplete, potentially affecting the accuracy of the findings. Second, the investigation focused on a subset of serum markers and endoscopic features as predictive indicators. Other potential biomarkers and clinical factors may also significantly influence the response to corticosteroid therapy, and their relevance warrants further evaluation. To strengthen the evidence base, further research should involve multicenter, prospective studies to confirm our findings and to identify additional predictive factors.

In conclusion, this study suggests that elevated serum IL-6 level is associated with poor response to corticosteroid therapy in patients with ICI-induced colitis. Although this finding highlights the potential of IL-6 as a predictive biomarker, it must be interpreted with caution given the single-center, retrospective nature of the study and the relatively small sample size. These limitations underscore the need for external validation and well-designed, multicenter prospective studies to confirm the predictive value of IL-6 and to determine its clinical applicability across diverse patient populations. Despite these constraints, our findings offer an important preliminary insight that could support earlier identification of patients at high risk for poor corticosteroid response. This, in turn, may guide timely therapeutic escalation, including the use of selective immunosuppressive therapies, thereby potentially reducing disease burden and improving patient quality of life. Importantly, such escalation does not appear to compromise overall survival outcomes. Thus, if validated in future studies, incorporating IL-6 measurement into clinical decision-making could facilitate a more stratified and personalized approach to managing ICI-induced colitis, ultimately optimizing treatment outcomes.

## Data Availability

The raw data supporting the conclusions of this article will be made available by the authors, without undue reservation.
