# Supplementary material for: Risk factors for steroid-refractory in immune checkpoint inhibitor-induced colitis: a retrospective cohort study
Source: Front Immunol. 2025 Oct 7;16:1623150. doi: 10.3389/fimmu.2025.1623150 (PMC12537882; doi:10.3389/fimmu.2025.1623150)
Supplement: Supplementary file 1 [file Table1.docx]

| **Supplementary Table 1. Univariate logistic regression analysis of risk factors associated with steroid refractory colitis** | | | | | |
| --- | --- | --- | --- | --- | --- |
| **Characteristics** | **Steroid-responsive group (n = 45)** | **Steroid-refractory group (n = 12)** | **OR** | **95% CI** | ***P* value** |
| **Age, median years (IQR)** | 61 (54-69) | 57 (52-67) | 0.979 | 0.921-1.042 | 0.510 |
| **Male, n (%)** | 32 (71.1) | 9 (75.0) | 1.219 | 0.284-5.232 | 0.790 |
| **BMI (kg/m^2^, mean ± SD)** | 22.0 ± 3.2 | 22.6 ± 2.4 | 1.061 | 0.867-1.299 | 0.566 |
| **Time to onset of colitis, median days (IQR)** | 97 (32-135) | 141 (79-184) | 1.011 | 1.001-1.022 | 0.037* |
| **Symptoms, n (%)** |  |  |  |  |  |
| Diarrhea | 44 (97.8) | 10 (83.3) | 0.114 | 0.009-1.380 | 0.088 |
| Blood in stool | 20 (44.4) | 8 (66.7) | 2.500 | 0.657-9.514 | 0.179 |
| Abdominal pain | 24 (53.3) | 8 (66.7) | 1.750 | 0.460-6.653 | 0.411 |
| Fever | 2 (4.4) | 3 (25.0) | 7.167 | 1.042-49.279 | 0.045* |
| **Tumor types, n (%)** |  |  |  |  |  |
| Gastrointestinal cancer | 21 (46.7) | 2 (16.7) |  |  | 0.416 |
| Lung cancer | 10 (22.2) | 5 (41.7) | 0.286 | 0.019-4.201 |  |
| Hepatobiliary cancer | 6 (13.3) | 3 (25.0) | 1.500 | 0.123-18.363 |  |
| Pancreatic cancer | 5 (11.1) | 1 (8.3) | 1.500 | 0.106-21.312 |  |
| Others | 3 (6.7) | 1 (8.3) | 0.600 | 0.027-13.582 |  |
| **ICIs types, n (%)** |  |  |  |  |  |
| Anti-PD-1 | 33 (73.3) | 8 (66.7) | 0.500 | 0.076-3.305 | 0.741 |
| Anti-PD-L1 | 8 (17.8) | 2 (16.7) | 0.462 | 0.056-3.811 |  |
| Combination (Anti-PD-1/L1 + Anti-CTLA-4) | 4 (8.9) | 2 (16.7) |  |  |  |
| **Number of ICIs treatment, median (IQR)** | 5 (2-8) | 6 (3-11) | 1.036 | 0.944-1.138 | 0.455 |
| **Diarrhea grade, n (%)** |  |  |  |  |  |
| 1 | 5 (11.1) | 2 (16.7) | 1.611 | 0.678-3.827 | 0.280 |
| 2 | 15 (33.3) | 2 (16.7) |  |  |  |
| 3 | 24 (53.3) | 5 (41.7) |  |  |  |
| 4 | 1 (2.2) | 3 (25.0) |  |  |  |
| **Colitis grade, n (%)** |  |  |  |  |  |
| 1 | 12 (26.7) | 1 (8.3) | 5.817 | 1.505-22.476 | 0.011* |
| 2 | 32 (71.1) | 7 (58.3) |  |  |  |
| 3 | 1 (2.2) | 1 (8.3) |  |  |  |
| 4 | 0 (0) | 3 (25.0) |  |  |  |
| **Laboratory results** |  |  |  |  |  |
| IL-6 (pg/mL) | 24.1 ± 20.5 | 81.7 ± 38.7 | 1.062 | 1.028-1.098 | ＜0.001* |
| Hb (g/L) | 110.3 ± 22.1 | 119.4 ± 17.2 | 1.022 | 0.989-1.057 | 0.193 |
| RBC (× 10^12^/L) | 3.6 ± 0.7 | 3.8 ± 0.5 | 1.843 | 0.647-5.253 | 0.252 |
| WBC (× 10^9^/L) | 5.9 ± 2.7 | 5.8 ± 2.3 | 0.982 | 0.766-1.259 | 0.885 |
| NEUT (%) | 60.9 ± 17.1 | 67.3 ± 10.4 | 15.372 | 0.184-1282.557 | 0.226 |
| PLT (× 10^9^/L) | 184.6 ± 91.0 | 180.9 ± 104.8 | 1.000 | 0.993-1.007 | 0.903 |
| ALT (U/L) | 23.3 ± 21.7 | 18.7 ± 10.9 | 0.984 | 0.942-1.029 | 0.481 |
| AST (U/L) | 28.3 ± 24.8 | 25.5 ± 12.1 | 0.994 | 0.962-1.027 | 0.705 |
| TP (g/L) | 63.9 ± 7.1 | 64.2 ± 8.0 | 1.006 | 0.921-1.099 | 0.888 |
| ALB (g/L) | 35.3 ± 5.2 | 37.4 ± 5.2 | 1.089 | 0.950-1.248 | 0.220 |
| ALP (U/L) | 112.3 ± 93.4 | 83.8 ± 24.7 | 0.993 | 0.979-1.007 | 0.317 |
| GGT (U/L) | 83.6 ± 104.4 | 61.8 ± 62.6 | 0.997 | 0.998-1.006 | 0.495 |
| TBil (μmol/L) | 10.6 ± 7.0 | 8.2 ± 3.1 | 0.909 | 0.771-1.071 | 0.255 |
| DBil (μmol/L) | 4.6 ± 5.1 | 3.0 ± 1.4 | 0.841 | 0.611-1.156 | 0.286 |
| BUN (mmol/L) | 5.2 ± 2.0 | 5.7 ± 2.5 | 1.116 | 0.838-1.486 | 0.452 |
| Scr (μmol/L) | 72.4 ± 16.3 | 81.8 ± 35.4 | 1.018 | 0.990-1.047 | 0.209 |
| LDH (U/L) | 176.8 ± 69.0 | 213.4 ± 72.2 | 1.007 | 0.998-1.016 | 0.121 |
| TT (s) | 17.5 ± 5.4 | 16.2 ± 1.1 | 0.797 | 0.495-1.283 | 0.351 |
| APTT (s) | 36.8 ± 8.3 | 34.1 ± 7.9 | 0.956 | 0.877-1.043 | 0.312 |
| PT (s) | 13.4 ± 1.5 | 12.9 ± 1.3 | 0.713 | 0.422-1.205 | 0.206 |
| PTA (%) | 91.6 ± 16.8 | 89.1 ± 9.6 | 0.989 | 0.947-1.032 | 0.608 |
| INR | 1.1 ± 0.1 | 1.0 ± 0.1 | 0.328 | 0.001-139.022 | 0.718 |
| D-dimer (μg/mL) | 1.8 ± 1.7 | 1.4 ± 1.0 | 0.817 | 0.491-1.358 | 0.435 |
| CRP (mg/dL) | 3.3 ± 3.9 | 4.6 ± 4.3 | 1.077 | 0.928-1.249 | 0.331 |
| **Endoscopic features, n (%) (n = 26)** | **n = 16** | **n = 10** |  |  |  |
| Ulcerative lesions | 7 (26.9) | 9 (34.6) | 11.571 | 1.172-114.262 | 0.036* |
| Non-ulcerative inflammation | 9 (34.6) | 1 (3.8) |  |  |  |
| Pan-colonic involvement | 8 (30.8) | 8 (30.8) | 4.000 | 0.639-25.020 | 0.138 |
| Partial colonic involvement | 8 (30.8) | 2 (7.7) |  |  |  |

ICI-induced colitis: Immune checkpoint inhibitor-induced colitis; IQR: interquartile range; SD: Standard deviation; BMI: Body mass index; ICIs: Immune checkpoint inhibitors; PD-1: Programmed cell death protein 1; PD-L1: Programmed death ligand 1; CTLA-4: Cytotoxic T-lymphocyte-associated antigen 4. IL-6: Interleukin-6; Hb: Hemoglobin; RBC: Red blood cell; WBC: White blood cell; NEUT: Neutrophil; PLT: Platelet; ALT: Alanine aminotransferase; AST: Aspartate aminotransferase; TP: Total protein; ALB: Albumin; ALP: Alkaline phosphatase; GGT: Gamma-glutamyl transpeptidase; TBil: Total bilirubin; DBil: Direct bilirubin; BUN: Blood urea nitrogen; Scr: Serum creatinine; LDH: Lactate dehydrogenase; TT: Thrombin time; APTT: Activated partial thromboplastin time; PT: Prothrombin time; PTA: Prothrombin activity; INR: International normalized ratio; CRP: C-reactive protein; OR: Odds ratio; CI: Confidence interval. * Represents statistically significant differences (*P* < 0.05).
